# Survey of Residency Directors’ Views on Entrepreneurship

**DOI:** 10.2196/19079

**Published:** 2021-04-14

**Authors:** Emily K Tam, Xuezhi Dong

**Affiliations:** 1 David Geffen School of Medicine University of California, Los Angeles Los Angeles, CA United States; 2 Boston Medical Center Boston, MA United States

**Keywords:** medical student education, medical student innovation, health innovation, program director

## Abstract

Medical students enter the medicine field with fresh ideas that may make them great entrepreneurs. However, medical students are uncertain about how the program directors of their desired residency would view them if they pursued business opportunities. We surveyed residency directors to obtain their views on medical students’ entrepreneurship experiences. This viewpoint article aims to help American medical students who are interested in health innovations understand how their interests and entrepreneurial experiences may affect how they are viewed by residency program directors. Most program directors had favorable views of medical students with experience in entrepreneurship, and they believed that the innovative traits gained from such experiences would add to the program.

## Introduction

Due to living in an era when innovative companies like Uber and Amazon are radically transforming the way we live our lives (from our transportation methods to our shopping methods), we are constantly exposed to new ideas that make life easier and more efficient. Despite people’s excitement for innovation, health care has been lagging in terms of adopting new ways to improve the health of Americans in a cost-effective manner [1]. In 2016, health care expenditures exceeded US $3 trillion in the United States, which is equivalent to US $9500 per person [2]. However, this amount of spending has not resulted in spectacular health outcomes, as the United States continues to have higher chronic disease rates; lower life expectancies; and poorer determinants of health, such as obesity, compared to other high-income nations [3,4]. One group in the medical profession that is beyond capable of being innovators in medicine is medical students.

Medical students enter the clinical medicine field with fresh and inquisitive minds [5]. Without years of experience and preconceptions, medical students can identify inefficiencies and challenges in the medicine field and have a strong desire to do something about them [5]. They often question the status quo of the health care system and ponder how it can be changed for the better. These characteristics have led to examples of successful companies started by medical students, such as Osmosis and SimX [6,7].

Although many medical students may have an interest in innovation and entrepreneurship, not many will actively pursue opportunities in these areas [8,9]. There is tremendous pressure for medical students to stay on the traditional pathway toward residency—obtaining glowing US Medical Licensing Examination scores, stunning clinical rotation evaluations, and prolific research achievements. Although health innovation is essential for improving the health care system, experts are unsure of how it can be integrated into medical training and, more importantly, how it affects students’ chances of being matched to their top choice residency programs [8,10]. To uncover how residency program directors perceive medical student entrepreneurship experiences in the application process, we conducted a survey of residency directors from some of the highest-ranked residency programs in the country.

## Methods

We sent a web-based survey via email to the directors of residencies across 16 different specialties that were affiliated with 17 top-ranked medical schools (according to the US News and World Report) [11] that represented the major regions of the country. The primary care–related fields that were represented included family medicine, internal medicine, obstetrics and gynecology, pediatrics, emergency medicine, and psychiatry. The nonprimary care–related fields that were represented included anesthesiology, radiology, neurology, general surgery, ophthalmology, orthopedic surgery, and plastic surgery. The initial email was followed by a reminder email that was sent approximately 1-2 weeks later.

The survey included both multiple-choice and open-ended questions. The multiple-choice questions included the following: (1) how many students with start-up experience did you encounter in the last 5 application cycles; (2) how does your program perceive students with start-up experience in the evaluation process; (3) what skills learned from start-ups do you believe can be applicable to a student training as a resident; (4) do you think using this time to work in start-ups or businesses would be beneficial for the student's clinical training; and (5) how would you rate your department/institution in terms of its receptiveness to new ideas? The multiple-choice responses were recorded on Google Forms and response percentages were calculated.

Open-ended questions included the following: (1) what advice do you have for medical students who are interested in entrepreneurship and start-ups; and (2) does your residency program permit students to take time off to pursue their research or academic interests? The responses to these questions were qualitatively analyzed by using a conventional content analysis approach, and notable comments are reported in the *Results* section [12].

## Results

We sent 190 survey requests; a total of 28 residency directors responded (response rate=15%). Of the 28 directors, 17 (61%) believed that providing start-up experiences in the residency application was favorable and increased the likelihood of being matched to a residency program. Further, 9 (32%) directors had neutral views on entrepreneurship experience, while 2 (7%) directors viewed the experience as unfavorable. All residency directors reported that they encountered medical students with entrepreneurship experience in the last 5 application cycles, with 10 (36%) reporting that they encountered 1-5 such applicants and 6 (21%) reporting that they encountered more than 15 such applicants.

When asked about what skills students can learn from start-ups that are applicable to residency training, 22 (79%) residency directors believed that students could gain communication skills, leadership skills, and the ability to innovate. Further, 20 (71%) surveyed directors believed that students could gain organizational skills, 18 (64%) believed that students could gain the ability to work in a team, and 16 (57%) said that students could gain better time management skills.

When residency directors were asked to rate their department or institution in terms of its receptiveness to new ideas, 13 (43%) directors reported that their institution was a very innovative place where new ideas were implemented rapidly, and 12 (46%) believed that their institutions were somewhat innovative and that new ideas could take some time to be implemented.

Although 24 (86%) residency directors reported that they permitted residents to take time off to pursue research or academic interests (duration was variable but could range from 6 weeks to 2 years), only 7 (25%) directors thought that taking time off to work in start-ups or businesses would benefit residents’ clinical training, 16 (57%) believed that such time off might help students, and 5 (18%) believed that such time off would not help residents.

Perhaps the more interesting insights came from the comments provided by the residency directors. Most comments revolved around the theme that students should focus on becoming great clinicians before pursuing entrepreneurial interests. A Johns Hopkins program director who viewed start-up experiences as favorable made the following comment:

Innovation in medicine is of the utmost importance...recently we have all expanded our view on how to fund and support new ideas. Start-ups are an excellent way to support innovation and we are all favorably inclined toward students with experience in this realm. The success or failure of the start-up is immaterial. The process itself is highly educational.

Another program director said:

I would encourage them, but to also reflect on what their ultimate professional goals are with a medical degree. Ideally, their experience would align with these goals. We look for this alignment in the application process.

A director also cautioned that “[it] is important to be up front with program directors regarding your interests.” They also stated:

Since you'll be matching into a job (as well as a training program) the program is expecting that your attention will be primarily on the training program so unforeseen changes in staffing can be disruptive. Talking in advance can help keep options open.

We also learned from directors who negatively viewed entrepreneurship. A director stated:

Wait until you are faculty. Our Program and others consider those students interested in entrepreneurship and start-ups to be unfocused, self-absorbed, and potential flight risks. While we interview students with such interests they definitely lose points when it comes time for ranking.

Another director said:

The only residents we've had quit our training program recently have been entrepreneurs. Despite the positive qualities inherent in an entrepreneur this has made us hesitant to match any more.

## Discussion

Despite our small sample size, our survey roughly gauged the opinions of directors of highly ranked residencies across multiple specialties in the United States. There was a diversity of opinions, but the majority of directors (17/28, 61%) perceived providing start-up experiences in the residency application as positive. Although they encouraged students to pursue entrepreneurial interests, residency directors almost unanimously believed that developing good clinical skills and becoming a good physician were the top priorities. There have been medical students who left their institution for a start-up before returning to school due to their desire to see patients again [13]. Since residency training is very demanding, many highly recommended students pursue other experiences before or after their residency rather than during their residency.

## Conclusion

We found that several residency directors were concerned that residents would quit their residency program to pursue other opportunities and therefore had more cautious attitudes. With regard to one’s career plans, clear and timely communication with residency program directors during the application cycle is crucial.
